# Using wearable devices for assessing the impacts of hair exposome in Brazil

**DOI:** 10.1038/s41598-019-49902-7

**Published:** 2019-09-16

**Authors:** Rodrigo De Vecchi, Júlia da Silveira Carvalho Ripper, Daniel Roy, Lionel Breton, Alexandre Germano Marciano, Plínio Marcos Bernardo de Souza, Marcelo de Paula Corrêa

**Affiliations:** 1 L’Oréal Research & Innovation, Rio de Janeiro, Brazil; 2 L’Oréal Research & Innovation, Clark, USA; 3 L’Oréal Research & Innovation, Aulnay-sous-bois, France; 4 Federal University of Itajubá (UNIFEI), Itajubá, Minas Gerais Brazil

**Keywords:** Environmental impact, Diagnostic markers

## Abstract

Previous studies have shown that physicochemical properties of hair can be impacted by internal and environmental exposures ranging from chemical stressors to weather. Besides the effects on hair, these exposures, termed “exposome”, can act on specific organs including skin, as a synergistic damaging effect of UV exposure and pollution on human surfaces. The combination of several environmental factors such as sun exposure, temperature, relative humidity, air pollution and photo-oxidation caused by ground level ozone impacts hair properties such as melanin oxidation, protein content, surface quality and structural components. Therefore, exposome studies can reveal new hypotheses on how epithelia and hair could be affected by daily life environment and routine. The aim of this study was to evaluate the impact of several environmental aggressors on human surfaces, using portable and wearable devices for monitoring exposome. To better understand the underlying mechanisms associated with environmental factors, two subjects wore multiple sensors to capture the meteorological conditions biking through urban areas in summer and winter. Temperature, humidity, UV radiation and ozone concentration were recorded and hair swatches of different types, including natural, bleached/colored, colored and gray, were exposed on the helmets. Silicon wristbands were used on skin to identify main chemical aggressors. After exposure, hair swatches were analyzed by surface microscopy analysis, oxidation markers and more than 1,500 chemicals were evaluated on the bracelets. Correlated with GPS and monitoring data, all these results provide insights on how environmental stressors affect the quality of different hair types and body surface according to exposure routine. Our results suggest extreme climate conditions associated with hair damage and photo-oxidative marker linked to the environmental aggressors. Polycyclic aromatic hydrocarbons (PAH) indicate possible causes of hair damages. This is the first meteorotropic study of its kind, combining environmental aggressors related to hair damage, opening new research hypothesis further studies on exposome.

## Introduction

Pollution and/or sun exposure are a global concern for human health, and the associated effects of environmental exposure on skin have been studied^1–4^. For instance, alterations in the epidermal and dermal structures through the chronological and photoaging processes are accelerated by environmental factors such as airborne pollutants, oxidative stress, lifestyles, diet, tobacco, personal care, hygiene and stress^5^. In a recent study, Krutmann and colleagues listed six major categories of compounds that can impact the skin status and aging: (i) sun radiations, such as ultraviolet radiation, visible light and infrared rays, (ii) air pollution, (iii) tobacco smoke, (iv) nutrition, (v) miscellaneous factors (stress, sleep, temperature) and (vi) misuse of cosmetic products. These factors cover what is now called the skin aging exposome^6^.

Ultraviolet solar radiation (UVR) and air pollution have been identified as aggressors to the human skin surface and deeper skin tissues. Beyond the carcinogenic effects of UVR (e.g., skin cancers), sun exposure is also responsible for skin ageing and hair damage^7^. More recently, epidemiological and mechanistic studies suggest that air pollution can also affect skin integrity^8^ and its combination with UV exposure can result in aggravated effects to skin health, since some pollutants (e.g., polycyclic aromatic hydrocarbons) are photo-reactive and phototoxic^4^.

It is likely that skin and hairs are exposed to significant concentrations of pollutants, either through systemic distribution or topical penetration of ultrafine particles. A high diversity of physical or chemical compounds can be classified as polluting agents, i.e. particulate matter (PM’s) of different sizes and with different chemical components and some gases, such as ozone and Nitrogen and Sulphur oxides. PM 2.5, composed by particles with a diameter <2.5 µm, are frequently associated with polycyclic aromatic hydrocarbons (PAHs), that can generate an oxidative stress when exposed to UVA radiation. Skin’s surface components can undergo an oxidation process, induced by some photo-reactive PAHs^9^. There is still no direct demonstration of the presence of pollutants within skin, but a previous work states that nanomolar PAH concentrations can be measured in blood^4^.

Other have demonstrated the ability of systemic PAHs to be transferred to human hair fibers. In this study, after washing the hair surface with appropriate solvents in order to eliminate external deposits of pollutants, parent molecules and hydroxy-metabolites of various PAHs were found in the inner layers of the hair (probably the cortex and medulla) at significant levels in the range of picomolar (average 118 pmol/g), suggesting that the biological incorporation of PAHs delivered by the blood may occur in human tissue surfaces, like the dermis and epidermis^10^.

Similar to skin, hair properties have previously been shown to depend not only on time (i.e., age) and intrinsic processes, but also on daily life conditions, such as nutrition, illness and stress, and the environment^11^. External impacts, including sun exposure, temperature, relative humidity can compromise hair growth and texture of the fiber^12^. Physical and chemical impacts on hair, by provoking lipid oxidation, tryptophan and cysteine degradation and breakage of disulfide bonds, can be designated as photo-aggravation of hair aging, which is characterized by an increase in hair porosity and surface roughness, and a decrease in mechanical resistance. After sunlight exposure, an increase in hair brittleness, dryness and stiffness can be observed^13^; the water-absorption capacity is also compromised. To avoid photo-aggravation effects, hair pigments act as a protection to the fiber, absorbing and filtering radiation and dissipating the energy as heat. In this process, pigments can be bleached and degraded. Eumelanin presents a higher photostability than pheomelanin, justifying why fair hair is not as resistant to photodegradation as dark hair^13^.

Environmental exposome can be defined as human’s airborne environmental exposure. The latter is majorly influenced by three variables: environmental conditions (as humidity, temperature, wind speed and PM density), spatial variables and lifestyle (location’s altitude, population density, and behaviors) and technical artifacts (batch effects)^14^. While exposome research has gained attention in recent years, there’s still no inexpensive and/or easy ways to evaluate the human exposome and the effects of environmental chemicals, including PAHs, on human health, skin and hair. There is an initiative to study human exposome using silicone wristbands and compare the results with validated and well-known assessment methods for PAH exposure. The study concluded that the wristbands can be considered a relevant tool to assess exposures and for use in environmental health studies^15^.

Hair swatches are another useful tool to directly assess how real-life environmental conditions can differentially impact varying hair types. In this paper, hair swatches were attached to cyclists in Rio de Janeiro/Brazil to evaluate how environmental conditions affect multiple hair types differently and illustrate how some hair types experience more damage than others. In the present study, meteorological instruments were used to record UV levels and urban pollutants and silicone wristbands were worn to measure chemical exposures when cycling through different routes in Rio de Janeiro. Hair samples were evaluated pre- and after post-environmental exposure using approaches such as Electron Microcopy and cysteic acid quantification to link people routine to environmental impacts on hair properties. This is the first meteorotropic study of its kind, combined with an exposome approach to assess the impacts on hair, generating relevant knowledge to create new hypotheses on environmental impacts due to exposition under extreme UV and climate conditions found in Brazil.

## Material and Methods

### Human hair swatches

Human natural (Virgin Brazilian Hair Type III, batch 102160, International Hair Importers and Products, Inc., Glendale, USA) and gray (Caucasian 90% Gray Hair, batch 1429BN90, International Hair Importers and Products, Inc., Glendale, USA) hair swatches (2.7 g/27 cm) were used in this study. Hair was classified according to the study conducted by Loussouarn and colleagues in 2007^16^. Prior to exposure, each hair swatch (2.7 g) was carefully washed in running water. A bland shampoo (1.08 g) was applied and scrubbed from the hair root to the hair tips using the index finger and the thumb five successive times. The hair was washed with running tap water, rubbing the swatch extension using the index finger and the thumb twenty times to guarantee that the shampoo would be completely rinsed off. The hair was dried with a blow dryer.

A subset of the natural hair swatches was affixed to a plastic clipboard involved with PVC and bleached. The bleaching solution was prepared with 25 g of a marketed bleaching powder and 50 g of a 30% volume hydrogen peroxide (H_2_O_2_) and mixed in a plastic hair coloring mixing bowl with a brush. In accordance with the product’s instructions for application, 27 g of the bleach mixture were applied immediately on the whole extension of one hair swatch, laid on one face, using the brush and gloved fingers and spread evenly over the entire swatch to guarantee that it would be homogeneously bleached on both sides. After the complete coverage of the hair swatch with the bleach solution was achieved, the swatch was wrapped into an aluminum foil for fifty minutes. The swatch was further washed with running water until the wash water was transparent. All swatches were washed with neutral shampoo and dried with blow dryer, following the steps previously described. A second bleaching process was conducted so that a lighter shade would be obtained.

A subset of the natural hair swatches and all of the bleached hair swatches underwent a coloring process in order to generate Colored and Bleached/Colored hair. For the coloring solution, 10.8 g of a coloring product (shade 6.11) and 16.2 g of a 20 volume H_2_O_2_ oxidant were mixed and applied on the hair swatches with a brush, following the same procedure for the bleaching. Thirty minutes post application, the swatch was completely washed with running water until the wash water was transparent. The hair swatches were washed with neutral shampoo and dried with a blow dryer, following the steps described above.

### Mobile meteorotropic measurements

**Figure 1:**
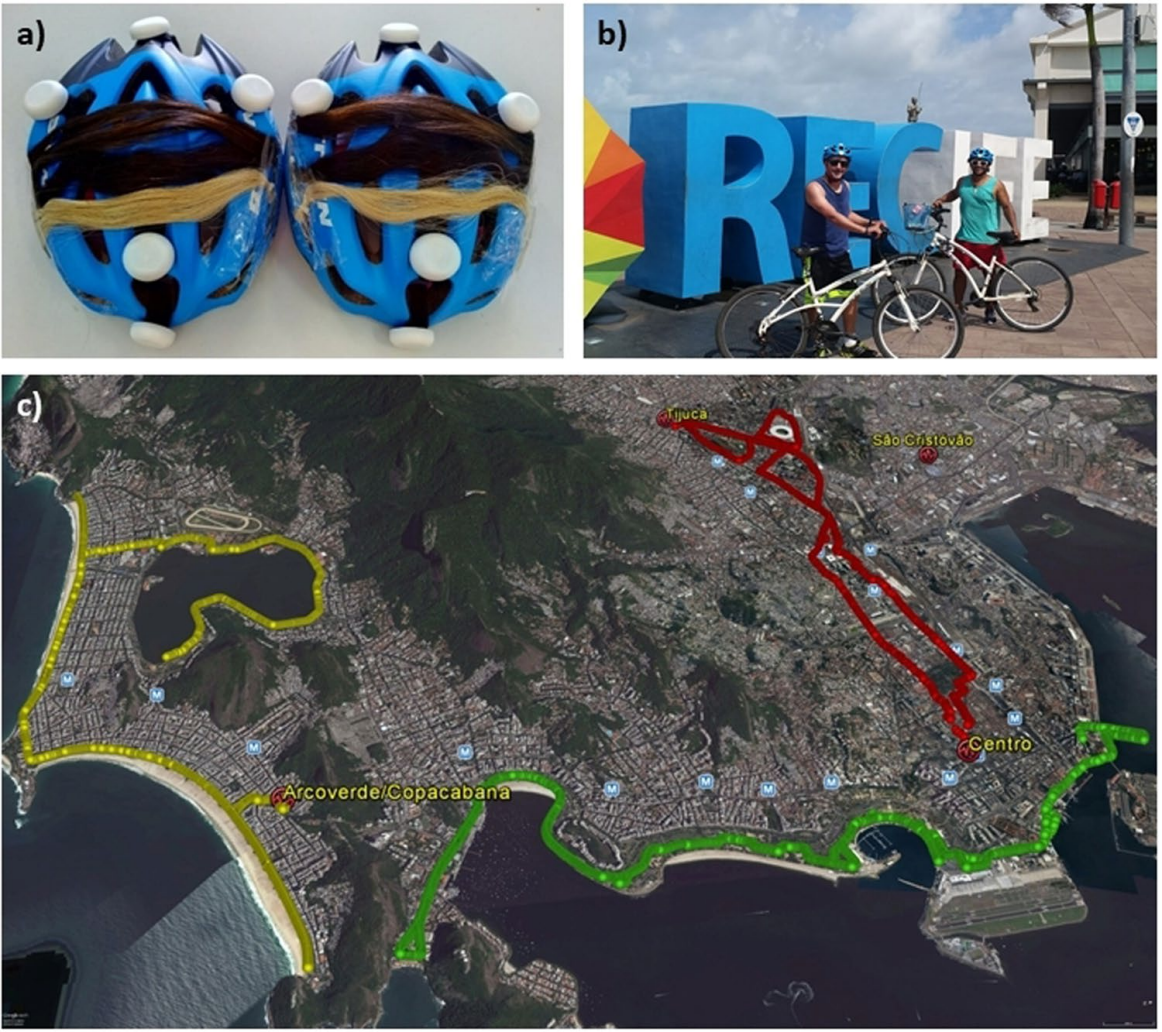
(**a**) Helmet with UV sensors and hair swatches (natural, colored, bleached/colored and gray). Fixed on the helmet. (**b**) Ordinary bikes adapted with scientific instruments. (**c**) Bike routes in Rio de Janeiro city in the experimental campaign of mobile measurement December 1^st^–3^rd^ 2016 (red route – day 1, green route – day 2, yellow route – day 3) – Image: Google Earth Pro V 7.3.2.5776. Rio de Janeiro, Brazil. 22.9 S, 43.2 W, Eye alt 10.0 km. Data SIO, NOAA, U.S. Navy, NGA, GEBCO © 2018 Google.

The measurements were conducted in Rio de Janeiro, Brazil (22.55° S; 43.10° W; 12 m) during three days in summer (December 1^st^–3^rd^, 2016) and in winter (July 6^th^–8^th^, 2017). We simultaneously measured erythemal UV doses (EryD), UV Index (UVI), surface ozone content (SOC), air temperature (T) and relative humidity (RH) using two bicycles and the helmets worn by the cyclists as mobile sampling platforms. UV-related measurements were performed by UV dosimeters (UV dosimeters badges, Scienterra Ltd, Oamaru, New Zealand) installed on the cycling helmets. Five of these electronic dosimeters were distributed on each helmet and the other instruments were attached on the bikes as can be seen in Fig. 1(a,b). A 2BTech Personal Ozone Monitor (Personal Ozone Monitor, 2B Technologies Inc, Boulder, USA - https://twobtech.com/pom-personal-ozone-monitor.html) was used for SOC samples; and Instrutherm thermo-hygrometers (Model HTR-170, Instrutherm Instrumentos de Medição, São Paulo, Brazil - https://www.instrutherm.net.br/termo-higrometro-digital-portatil-com-saida-rs-232-e-datalogger-mod-htr-170.html) performed T (°C) and RH (%) measurements. Designated cycling routes were chosen for covering touristic or urban crowded sites in tours of around 20–25 km per day (Fig. 1(c)). In general, these tours began between 30 min and 1h30 before noon with data sampled every 10 seconds.

Some of the hair swatches were attached to the cycling helmets and underwent an environment exposure. Those samples were classified as exposed hair swatches. The other hair swatches were classified as the unexposed ones, as controls.

### Exposome analysis

Wristbands (MyExposome, Inc, Philadelphia, PA) were worn by each cyclist to collect environmental samples^17^. The participants wore wristbands for three consecutive days during summer, cycling for 6 hours per city. 2 hours per day in average, during 3 sequential days in each city, depending on the weather conditions. All measurements were performed in duplicates (n = 2 cyclists). At the end of sampling campaign, all samples of hair swatches and bracelets exposed during outdoors experimental campaigns were labeled and stored in air tight polytetrafluoroethylene (PTFE) bags (Welch Fluorocarbon,Dover, NH, USA) and stored at −20 °C until analysis After deployment, field staff shipped the wristbands to Oregon State University (OSU) in PTFE bags. The wristbands were cleaned twice with 18 MΩ cm water and once with isopropanol to remove particles on the surface. The wristbands were immediately stored in amber jars at −20 °C until extraction. Briefly, each was spiked with extraction surrogates to account for extraction efficiency, then extracted twice in 100 mL ethyl acetate at room temperature using an orbital shaker set at 60 rotations per minute and quantitatively concentrated using TurboVap® closed cell evaporators (Biotage LLC, Charlotte, NC, USA). All of the sample collections took place in 2016^15^.

Each wristband was analyzed and screened for 1,529 compounds as described previously^18^. Every chemical in the testing is classified into one or more of the categories presented in Table 1. The list was achieved by adding approximately 450 chemicals to libraries purchased with the deconvolution software. Single component standards (analytical grade) were prepared from neat or purchased in ethyl acetate, methylene chloride, n-hexane, or isooctane at a concentration between 0.5 and 10 ng/μL. Mass spectra and retention time for each new chemical were acquired using the GC-MS method described above and added to a new ChemStation probability-based matching library. Each entry included chemical name, retention time, retention index (retention time in seconds), mass spectra, molecular formula (from which the software generates MW) and Chemical Abstracts Service registration number (CASRN). After new chemicals were added, the new library was appended to the master library. AMDIS library files and an updated method file were then generated from this master library using ChemStation software^16^.

**Table 1:** Wristband chemicals classification and description.

Classification	Classification Description
Chemicals in Commerce	Chemicals found in consumer industrial, or commercial products or products streams.
Consumer Products	Chemicals found in foodstuffs or other consumable goods, such as cigarettes, coffee, and spices or other products intended for household or personal use.
Dioxins and Furans	Most dioxins and furans are not man-made or produced intentionally but are created when other chemicals or products (such as herbicides, pulp, paper) are made. In addition, they can be produced when products are burned.
Flame Retardant	Flame retardants used in consumer and commercial products such as polybrominated diphenyl ethers (PBDEs), polybrominated biphenyls (PBBs), and organic phosphate flame retardants (OPFRs) and others.
OPAH	Oxygenated Polycyclic Aromatic Hydrocarbons (OPAHs): found in fossil-fuel/combustion-based sources or weathered PAHs.
PAH	Polycyclic Aromatic Hydrocarbon (PAHs): found in petroleum, fuels and combustion of organic matter.
Personal Care	Found in many personal care products (shampoos, perfumes, other cosmetics).
Pesticide	Herbicides, fungicides, insecticides, rodenticides, etc. or degradation products of pesticides.
Pharmaceutical	Used in making or as components of manufactured drugs.
Polychlorinated Biphenyl	Manufactured chlorinated chemicals that are found in electrical, lighting, gas and construction industries.

### Scanning electron microscopy

Scanning electron microscopy (SEM) was used to assess the environmental damage on hair swatches^19^. Only hair swatches from the summer seasonal study underwent a SEM analysis. Generally, damaged hair samples present broken edges, holes, cracks and scratches, particles deposited on the surface and some detached and dented cuticles due to environmental damage. The images of hair damage were obtained using the high-resolution Scanning Electron Microscope Phenom ProX, with a magnification of 2,200×. Exposed and unexposed hair fibers were fixed on the specimen stub using carbon adhesive and had their length scanned. At least 10 images of non-overlapping regions were obtained from each of 3 hair fibers for each one of the 12 hair swatches.

### Cysteic acid quantification

For the quantification of cysteic acid, the hair swatches from the summer study were named with the following codes: NO for Natural Hair Unexposed, N1 for Natural Hair Exposed, CO for Colored Hair Unexposed, C1 for Colored Hair Exposed, DCO for Bleached/Colored Hair Unexposed, DC1 for Bleached/Colored Hair Exposed, GO for Gray Hair Unexposed and G1 for Gray Hair Exposed. The spectroscopies were made in triplicate using Infrared spectrophotometer (Model Frontier - PerkinElmer) with ATR cell (Pike Technologies) and ZnSe crystal.

### Statistical analysis

Graph results are presented as box plots, using 25^th^ (lower quartile or Q1), 50^th^ (median or Q2) and 75^th^ (upper quartile or Q3) percentiles and the interquartile region (IQR = Q3 – Q1) to describe a sample with a confidence interval of 95%^20^. Differences were tested for significance as indicated in the figure legends. Statistical tests were performed with Graph Pad Prism 6.

## Results and Discussion

### Meteorological data

**Figure 2:**
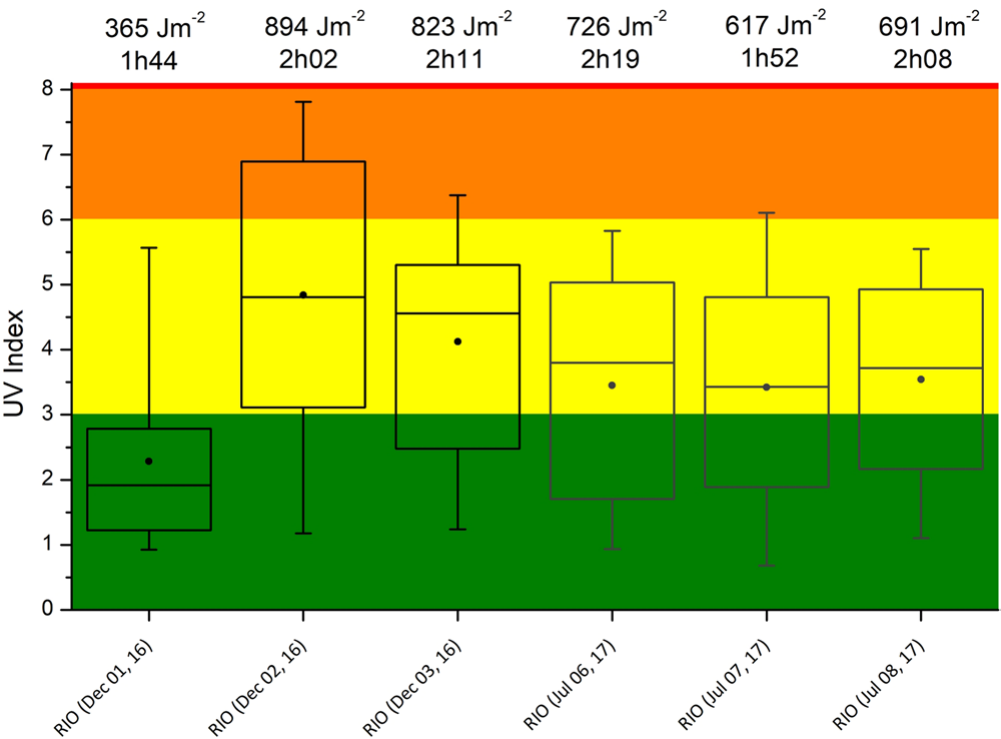
Boxplot of UVI measured during experimental campaigns in Rio de Janeiro in summer and winter. Vertical lines show the 5–95 percentile intervals, dots are the mean and horizontal lines inside the boxes are the medians of UVI. UVI colors give an indication of the level of UV radiation and the potential danger of sun exposure: green (low), yellow (moderate), orange (high), red (very high) and violet (extreme).

In this study, the combination of UV exposure and pollution was correlated with the impacts caused in different hair types and treatments, looking at hair surface quality and specific photo-oxidative markers within the hair cortex. The accumulated UV erythemal doses (AED) and the exposure time of the hair strands are shown in Fig. 2, which also shows a summary of UV Index (UVI) data collected in both campaigns. The average exposures for 5 of the 6 experimental campaigns suggest moderate potential danger of sun exposure. The seasonal UV doses differences were smaller than expected and not statistically significant. This may be due to the presence of clouds, as the three summer tours were performed on cloudy days. This is not unexpected, as summer is a rainy season and solar radiation can be attenuated in cloudy days. On the other hand, wintertime is the driest season, with cloudless skies and sunny days.

Despite cloudy conditions observed in most of summer measurements, AED reached MED for phototype V in all days, with the exception of one rainy day (December 1^st^). Winter measurements show similar results with significant UVR reaching the surface. These results indicate a relevant potential damage for the human hair. It is important to clarify that we have used the skin erythemal photobiological response as a parameter for the hair damage effects. As for the skin erythemal response (redness), UVB range is more harmful than UVA for hair damage. Once the main chromophores of hair proteins absorb the UVB wavelengths, UVA acts as a secondary cause of damage.

**Figure 3:**
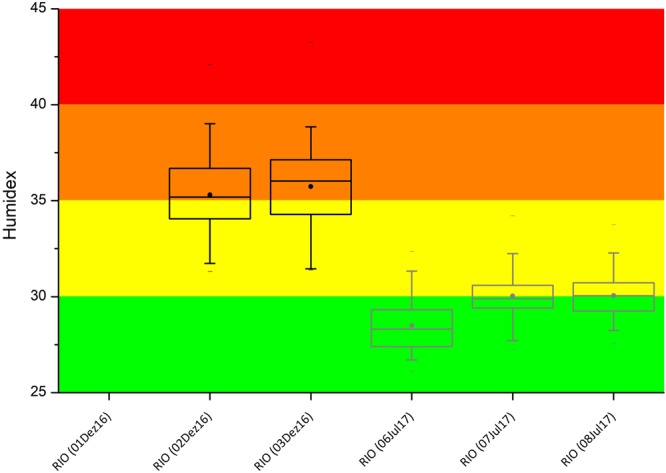
Humidex index in Rio de Janeiro during Meteorotropic experiment. Vertical lines show the 5–95 percentile intervals, dots are the mean and horizontal lines inside the boxes are the medians of Humidex. Black and gray boxes indicate summer and winter measurements, respectively. Colored stripes show comfort levels. Green: H < 29 – Little or no discomfort; Yellow: 30 < H < 34 – Noticeable discomfort; Orange: 35 < H < 39 – Evident discomfort; and, Red: 40 < H < 45 – Intense discomfort, avoid exertion. Data from Dec 01, 16 could not be recorded due to weather conditions.

On average, T °C and RH% measurements also showed typical values for both seasons. T °C ranged between 25 °C and 34 °C in summer, and between 22 °C and 29 °C in winter. RH% ranged between 50% and 80% in both seasons. Humidex – a thermal comfort index related to these measurements – is shown in Fig. 3. Humidex combines these two meteorological parameters into one number to reflect the perceived temperature for comfort evaluation. A severe thermal discomfort was observed during the summer campaign and noticeable discomfort could also be perceived in wintertime. These results do not mean significant risk of succumbing to the serious health or heat illness. However, the thermal discomfort can cause sweating and may lessen the efficiency of sunscreens. In addition, it is common to report the discomfort in the use of sunscreens in extreme heat situations.

**Figure 4:**
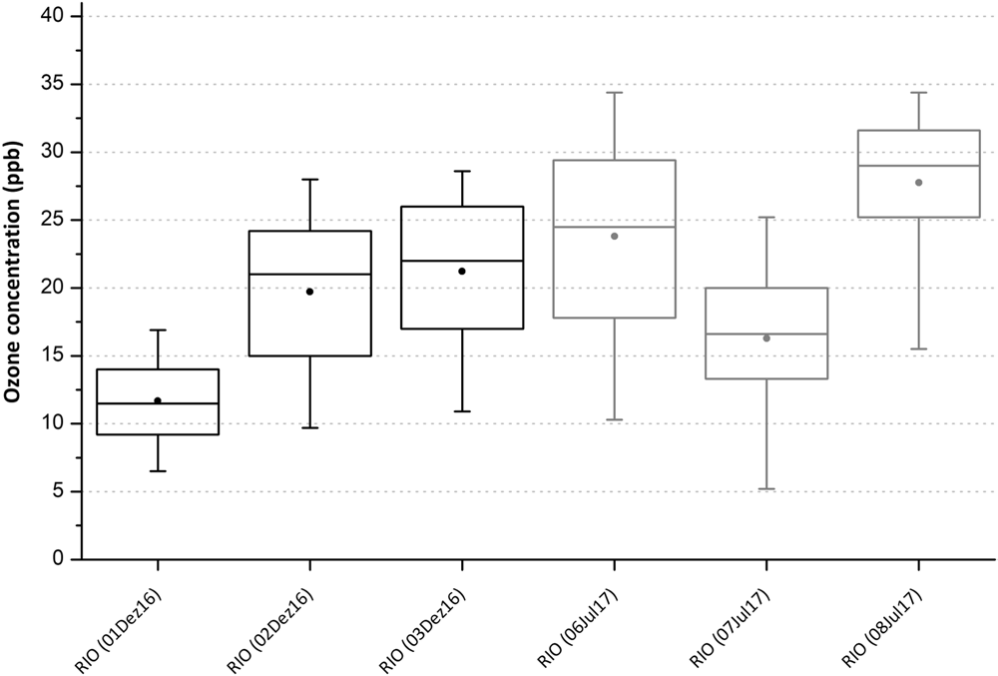
Surface ozone concentration in Rio de Janeiro during the Meteorotropic experiment. Vertical lines show the 5–95 percentile intervals, dots are the mean and horizontal lines inside the boxes are the medians of surface ozone concentration. Black and gray boxes represent summer and winter measurements.

Lastly, Fig. 4 shows Surface Ozone Concentration (SOC) measurements. SOC ranged between 12 ± 4 to 28 ± 6 ppb during the campaign. Recent air quality standards regulated by US Environmental Protection Agency^21^ considers ozone standard levels to 70 parts per billion (ppb) in averaging times (eight hours). These levels are the same standards recommended by the Brazilian Ministry of Environment^22^. European standards^23^ are more restrictive with target values of 60 ppb in 8-hour mean. Rio’s SOC measurements during both campaigns were under the 50 ppb of SOC recommended by the World Health Organization (WHO). Therefore, ozone cannot be considered as a strong source of pollution in Rio de Janeiro.

### Hair samples

Natural, colored, bleached/colored and gray hair swatches were exposed to environmental conditions, for approximately 2 hours per day for 3 days. This seasonal study considered summer impacts on hair fiber conditions. SEM was used to observe physical damages on hair after outdoor exposure.

All hair fibers contained some broken edges, holes, cracks, scratches and particles deposited on the surface. Importantly, there were no major differences in the quality of the hair fiber following environmental exposure, with the lone exception of the gray hair swatch, which exhibited detached cuticles and revealed cortex post-exposure (Supplementary Fig. 1). There are also chemical changes that could result from environmental exposure, such as lipid oxidation, tryptophan degradation and disulfide bond cleavage.

**Figure 5:**
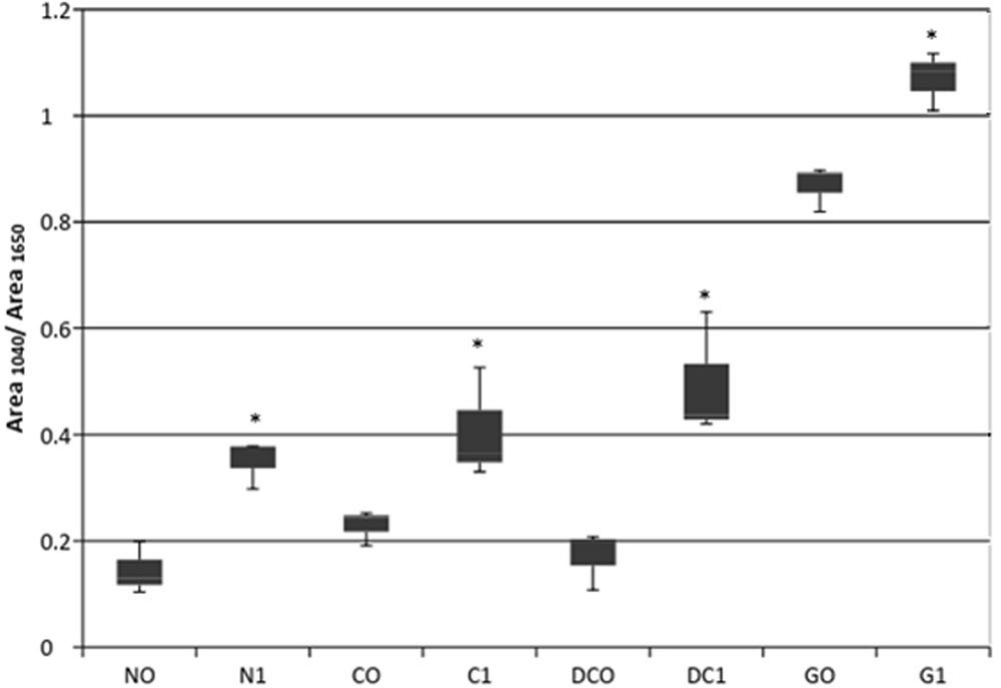
Quantification of Cysteic Acid on hair surface in NO (Natural Unexposed), N1 (Natural Exposed), CO (Colored Unexposed), C1 (Colored Exposed), DCO (Bleached/Colored Unexposed), DC1 (Bleached/Colored Exposed), GO (Gray Unexposed) and G1 (Gray Exposed) Hair Swatches. The exposed and unexposed hair swatches present significant difference (*relates to p < 0.05) as well as the groups when compared with one another.

Studies have shown that, as a consequence of UV radiation exposure, it is possible to observe amino acids degradation and oxidization of melanin and internal lipid in irradiated hair swatches, weakening the fiber’s structural integrity^17^. Further, hair damage has been shown to occur through cysteic acid and free radicals following UV-induced disulfide bond breakage^24^. Therefore, cysteic acid was quantified as a potential marker to analyze the effect of environmental aspects of hair. Despite the minimal observable physical damages to the hair fiber, there was a significant increase, at different levels in cysteic acid quantified on the fiber surface after environmental exposure, for all tested groups (Fig. 5).

It is known that UV radiation is the main contributor to oxidative damage on hair, which leads to hair’s color fading and dulling. Photo-oxidation of amino acids and fatty acids, sulfur bridges cleavage and lipids decomposition are some consequences of UV exposure that induce hair damage^25^. It is observed that the bleached/colored and the gray hair swatches present more physical damage and cysteic acid content following exposure in comparison to the natural and bleached hair, potentially due to the amount and type of hair pigments in the hair samples. Melanin has a photo-protective effect on hair, absorbing and filtering radiation and dissipating the energy as heat. Both types of natural hair pigments, pheomelanin and eumelanin, are degraded or bleached while protecting the hair. Pheomelanin has a lower photostability than eumelanin and, since darker hair has a higher content of eumelanin when compared to lighter hair, it is more resistant to photodegradation. This degraded melanin is one potential explanation for why the bleached hair swatches presented more damage than the natural hair and the colored ones. Similarly, gray hair has no pigment and, because of that, is the less protected. Consequently, it is shown to be the most damaged hair swatch after environmental exposure.

However, there are environmental reactive species which could also impact hair quality. Ground level ozone uptake rate by human hair has been already quantified, and the resulting formation of volatile aldehydes and ketones was determined. Volatile reaction products consistently observed include geranyl acetone and 6-methyl 5-hepten 2-one, as reaction products between ozone and squalene. Compounds formed as a result of ozone chemistry with personal care products have not yet been identified. On average, the integrated ozone uptake for the washed hair samples was 1.12 × 10^−5^ mol O_3_ g^−1^ and for unwashed hair was 1.87 × 10^−5^ mol O_3_ g^−1^. For a hair sample that was not washed for 3 days ozone uptake was about ten times higher than the average for all other hair samples. Further the yield of geranyl acetone, 6 methyl 5-hepten-2-one and decanal was consistently higher on unwashed hair. While natural oils that coat hair contribute to ozone reactions on unwashed hair, ozone appears to be reacting with other, unidentified compounds on washed hair^26^.

### Exposome

**Figure 6:**
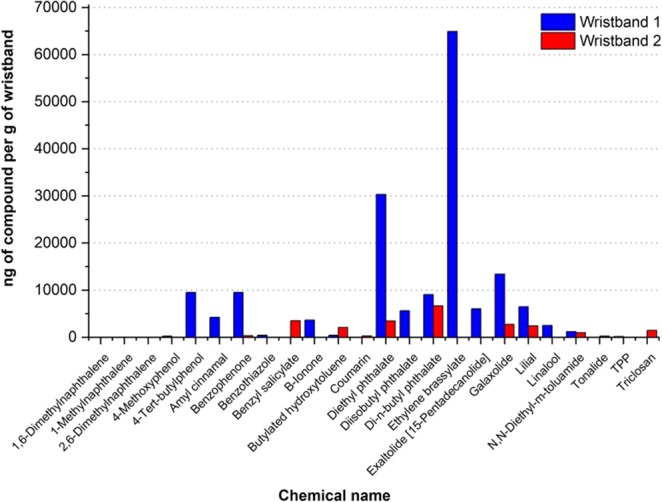
Quantification of chemicals found in the Exposome wristbands in nanograms in each gram of wristband (daily averages).

Personal exposome is dynamic and varies spatiotemporally^14^. In the present study, differences between riders’ exposomes depend on airborne pollutants, such as naphthalene and derivatives, and mostly on hygiene, pharmacological, personal care and fragrance products used by the individual participants. Most of the compounds found in our study were from personal care and consumer products. The majority are used as flavorings, fixatives and fragrances in soaps, detergents, shampoos, perfumes, deodorants, laundry and dishwashing products. Others are found in pesticides, repellents and flame retardants. But, some of the compounds are identified as PAHs, found in petroleum, fuels and combustion of organic matter. PAHs are classified as environmental pollution and designated as biomarkers of pollutants exposure^27^. In this study, PAH levels were considered as the main pollution marker, since there are synergic effects with PAHs and other chemicals resultant from Brazil’s extreme conditions regarding UV levels, temperature and humidity (Fig. 6).

As shown in Fig. 6, the two participants present different exposomes, regarding which compounds were found in the wristbands and their concentrations. Table 2 presents the classification of the compounds found in the two wristbands under analysis.

**Table 2:** Chemicals Classification.

Chemical Name	Classification
1-Methylnaphthalene	PAH, Chemicals in Commerce
1,6-Dimethylnaphthalene	PAH
2,6-Dimethylnaphthalene	PAH
4-Methoxyphenol	Pharmacological, Chemicals in Commerce
4-Tert-butylphenol	Chemicals in Commerce, Consumer Products
Amyl cinnamal	Personal Care
B-Ionone	Personal Care
Benzophenone	Personal Care, Chemicals in Commerce
Benzothiazole	Chemicals in Commerce
Benzyl salicylate	Personal Care
Butylated hydroxytoluene	Chemicals in Commerce, Consumer Products
Coumarin	Personal Care, Consumer Products
Di-n-butyl phthalate	Personal Care, Chemicals in Commerce, Pesticide
Diethyl phthalate	Chemicals in Commerce, Pesticide
Diisobutyl phthalate	Chemicals in Commerce
Ethylene brassylate	Personal Care
Exaltolide [15-Pentadecanolide]	Personal Care
Galaxolide	Personal Care, Chemicals in Commerce
Lilial	Personal Care
Linalool	Personal Care, Pesticide
N,N-Diethyl-m-toluamide	Pesticide
Tonalide	Personal Care
TPP	Flame Retardant, Chemicals in Commerce
Triclosan	Pharmacological, Personal Care, Chemicals in Commerce

In average, 16 chemicals were found across all tested wristbands in this project, at maximum 19, 13 at minimum on one wristband. The standard deviation of the number of found chemicals per wristband was 3.01. Considering both bracelets, a total of 24 distinct chemicals were detected in Rio de Janeiro city.

**Figure 7:**
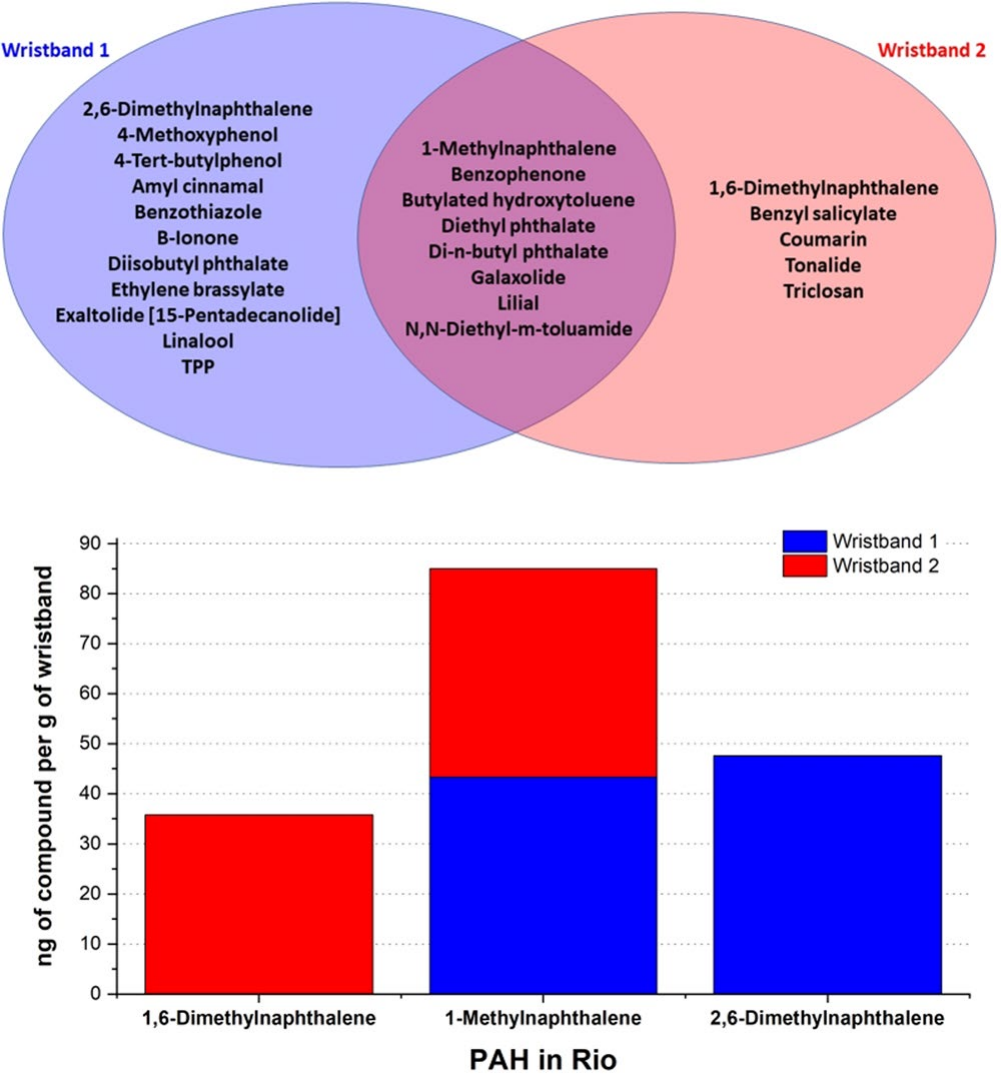
(**A**) VENN Diagram comparing the wristbands’ composition. (**B**) Chart comparing the daily exposure in nanograms/day for each PAH found in Rio de Janeiro, summing the quantities found in both wristbands.

Of particular interest to this study are PAHs, as of environmental concern, presenting toxic, mutagenic and/or carcinogenic properties, and are promptly absorbed by the gastrointestinal tract of mammals, since highly lipid soluble, and rapidly distributed in a wide variety of tissues^28^. Figure 7(A) compares both of the participants’ exposomes, showing the common and different compounds found in the wristbands. Of note, 3 PAHs were detected in this study, with 1-Methylnaphthalene detected in both wristbands. Figure 7(B) shows the participant’s daily exposure to PAHs in nanograms/day.

Unlike the compounds associated with personal care products, which result from direct contact with the bracelet, are specific to the wrist location and therefore unlikely to affect the hair, PAHs are present in the environment. Thus, it is likely that the relative levels of PAHs detected in the wristbands are representative of the levels of hair exposure. PAHs adhere on the hair surface and the toxic and oxidizing pollutants penetrate inside the hair fibers when the pollutants are dissolved in liquid droplets (oil) or water (humidity). Pollutants are responsible for chemical damage to hair cuticle and protein, sometimes irreversible, leading to worse conditioning and more hydrophilic hair^29^.

The silicon bracelets have properties that allow the collection of a wide range of chemicals, including pollutants and daily products that could accumulate. A recent study performed in China measured the presence of chemical components directly on the hair and observed concentrations of PAHs similar to the levels found in our analysis^27^. Although the physicochemical composition of hair and the silicon bracelets are different, the ambient demonstration, occupational application and statistical significance were previously demonstrated^17^, and our experiments followed the same Quality Control protocol. The PAH levels found in our study were comparable and appear in the same order of magnitude and ratio between them. It suggests that simple wearable devices could be an indicator of accumulated impact of pollution on hair, but for more precise measurements, other sensible quantification techniques such GC-MS should be deployed directly on hair swatches to quantitative measurements.

Through the exposome analysis, it could be concluded that both riders were exposed to different compounds due their personal care and hygiene routines, which resulted in the majority of the components being identified as fragrances and fixatives. The PAHs found were classified as pollutants and can be designated as one of the causes of hair damage.

The present study is unique in its ability to capture and quantify real-life exposure conditions and the corresponding chemical effects these exposure conditions exert on hair fibers. While there are other factors that contribute to the damaging effects of environmental exposure to the hair swatches that were not explicitly measured in this study (e.g., wind), this study is the first (to our knowledge) to connect PAH identification and levels with hair damage under real-life circumstances. It is of particular interest that the increased levels of cysteic acid were observed after such short exposure times (approximately 6 hours), which in turn, motivates future work to identify the effects of prolonged environmental exposure. We acknowledge that in order to make conclusive statements regarding the exact cause(s) of the hair damage, further work is needed to expand the study population, and vary the PAH exposure levels and meteorological conditions. Nonetheless, the work presented here, while correlative, paves the road for better identifying causative factors involved in hair damage.

## Conclusion

In the present study we correlate the photo-pollution exposure in different hair types and treatments to specific photo-oxidative markers within the hair cortex. This is the first meteorotropic study of its kind, combined with an exposome approach to assess the impacts on hair, generating relevant knowledge to create new hypotheses on environmental impacts due to exposition under UV and climate conditions found in Brazil. This knowledge was supported using instrumental evaluation to assess hair damage markers and scanning electron microscopy to demonstrate hair quality parameters. Our main conclusions are based on extreme climate conditions in Brazil. Our data show that, under such severe conditions, the hair is quickly damaged, evidenced by an increase in cysteic acid following only 6 hours of environmental exposure, independently of hair type. Colored, bleached/colored and gray hair are more impacted by environmental aggressors, such as UV and pollution, which are the main perceived aggressors on Brazilian consumer hair. Similar to skin, the degradation of hair proteins is likely caused by oxidative stress, in different levels and hair layers, according to UV wavelength and penetration potency. Here several photo-oxidative markers were investigated and cysteic acid appeared as a potential discriminative marker for the hair damage specifically caused by environmental aggressors, such as UV, pollution and exposome.

The knowledge generated during this work can support future studies, suggesting potential targets related to hair damage, to be validate and used to develop new evaluation methods with controlled conditions, as well as applied in preventive health exposome approaches.
